# Evaluation of Pollutants Along the National Road N2 in Togo using the AERMOD Dispersion Model

**DOI:** 10.5696/2156-9614-10.27.200908

**Published:** 2020-08-25

**Authors:** Yawovi Mignanou Amouzouvi, Milohum Mikesokpo Dzagli, Koffi Sagna, Zoltán Török, Carmen Andreea Roba, Alexandru Mereuţă, Alexandru Ozunu, Kodjovi Sidéra Edjame

**Affiliations:** 1 Department of Physics, University of Lomé, Lomé, Togo; 2 Faculty of Environmental Science and Engineering, Babeş - Bolyai University, Cluj-Napoca, Romania; 3 Department of Geography, University of Lomé, Lomé, Togo

**Keywords:** air pollution, environment, road traffic, AERMOD, gas emission, Togo

## Abstract

**Background.:**

Air pollution has become a major problem around the world and is increasingly an issue in Togo due to increased vehicular traffic. Gaseous pollutants are released by engines and are very harmful to human health and the environment. The fuels used on the major road in Togo, the N2, are adulterated with unknown contents and are of poor quality. Many of the vehicles come from neighboring countries, such as Benin, Ghana and Nigeria.

**Objectives.:**

The present study aims to evaluate the pollution rate in Togo through the estimation of the concentrations of sulfur dioxide (SO_2_), nitrogen oxides (NO_x_), and particular matter (PM) on the international road, the National Road N2, in Lomé, compared to the World Health Organization's (WHO) standard limit.

**Methods.:**

The simulations of pollutant concentration were performed using the Industrial Source Complex Short Term Version 3 model, which is included in the United States Environmental Protection Agency Regulatory Model (USEPA) AERMOD View software. The meteorological averages data were obtained from the local station near the National Road N2 in Togo in 2018. Hourly averages were calculated according to the European Monitoring Evaluation Programme/European Environmental Agency air pollutant emission inventory guidebook 2016 and were processed using AERMET View and a terrain pre-processor, AERMAP. For the model, the sources of pollution were the vehicles traveling on the road segment. The source was a line volume with 20 m of width and 2 m of height. The estimation methodology covered exhaust emissions of NO_x_, SO_2_ and PM contained in the fuel.

**Results.:**

The simulations provided average hourly, daily and annual concentrations of the different pollutants: 71.91 μg/m^3^, 42.41 μg/m^3^,11.23 μg/m^3^ for SO_2_; 16.78 μg/m^3^, 9.89 μg/m^3^, 2.46 μg/m^3^ for NO_x_ and below the detection limit, 0.62 μg/m^3^, 0.15 μg/m^3^ for PM, respectively. These results indicate that on the National Road N2 in Togo, the concentrations of SO_2_ were high compared to those of NO_x_ and PM. The daily average concentration of SO_2_ was twice the permissible limits set by the WHO.

**Conclusions.:**

Emissions obtained from the AERMOD for NO_x_ and PM were less than the permissible limits set by the WHO, while the rate of SO_2_ was twice the permissible limit. The fuels used on this road were very rich in sulfur. The sulfur level in fuels must be monitored by stakeholders in Togo.

**Competing Interests.:**

The authors declare no competing financial interests.

## Introduction

Atmospheric pollution refers to the presence of pollutants (gaseous or particles) in the atmosphere. Air pollution can have harmful effects on the environment and human health.^1^ The sources of this pollution can be either natural or related to human activities, especially combustion processes (motor vehicles, industrial installations, energy production, etc.).^2^,^3^ Many studies have shown a correlation between the degradation of the environment and human health and the presence of pollutants in the atmosphere.^4^ Exposure to air pollution is responsible for respiratory and lung diseases and leads to premature deaths worldwide (4.2 million in 2016 according to the World Health Organization (WHO)).^5^–^7^ Pollution contributes to climate change and the phenomena of acid rain which has a very harmful effect on vegetation and global warming.^1^,^8^ Sulfur dioxide, emitted from fossil fuel consumption or industrial activities, is an acidifying gaseous pollutant that forms sulfuric acid in the presence of water. It contributes to the phenomenon of acid rain that disrupts the composition of air, surface water and soil, and could damage plants and vegetation and kill animal species.^1^,^9^ At higher concentrations, sulfur dioxide can have serious health effects and impact pulmonary function. Apart from industrial processes, road traffic is an important source of atmospheric pollution in the world, especially in developing countries, due to the age of vehicles used and poor quality of fuel.^10^ Nitrogen oxides constitute very toxic odorant gases, and are formed by the oxidation of nitrogen in the air, from fuels with oxygen or during the combustion phenomena in engines. They have harmful effects on human health and the environment with the formation of ozone (related to the greenhouse effect), and increased sensitivity of the bronchi to microbial infections for children.^11^ For nitrogen dioxide (NO_2_), a traffic-related pollutant, short-term exposure causes significant inflammation of the respiratory tract, reduces lung function and increases symptoms of bronchitis in those with asthma.^7^

Particulate matter (PM) is comprised of ultrafine particles that impact human health. These small aerosols can penetrate deep into the lungs and into the alveoli.^12^,^13^ Nuclei condensation can be formed where moisture and pollutants (lead, sulfur dioxide) can absorb, making them even more toxic. Therefore, PM is an important vector of respiratory tract intoxications in areas of high traffic.^14^ Diallo and coworkers demonstrated the correlation between air quality and its impact on respiratory diseases due to PM in Lomé, Togo.^15^ Air pollution in urban areas due to road traffic is an important issue in developing countries. Globally, many countries have little or no access to low-sulfur fuel, and do not have standards for vehicle emissions.^16^ The sulfur content in fuel in most developed countries is currently 50 ppm sulfur or less; however high sulfur content can be found in many low- and middle-income countries from ranges of 500–5000 ppm.^16^ Transport traffic is estimated to grow rapidly by 2050, which will double global fuel demand.^17^ Almost 50% of the fuels imported to West Africa come from Amsterdam, Rotterdam and Antwerp, and trade statistics showed that 80% of the diesel exported to Africa has a sulfur content at least 100 times above the European standard.^18^

**Abbreviations**
PM: Particulate matter
UTM: Universal Transverse Mercator
WHO: World Health Organization

In Togo, more than 50 000 motorcycles and taxis travel on traffic roads daily, polluting the atmosphere, and as a result the population has respiratory problems due to the poor quality of fuels that these vehicles consume.^19^ The N2 is an international road not only used by vehicles from Togo, but cars and buses from Nigeria and Benin travel through to Togo, Ghana and the Côte d'Ivoire and vice versa. In Togo and along the National Road N2, even in Benin and Nigeria, the most used fuels are adulterated with unknown contents of sulfur (locally called “Boudé” or “Kpayo” in Togo and Benin). It is thus necessary to determine air pollution in this area.

Five West African countries including Togo introduced standards to regulate emissions and lower the levels of sulfur diesel in their fuels in 2016.^18^ Many studies have been carried out in countries using AERMOD simulation for the measurement and control of air quality and assessment of their impact on human health and the environment.^20^–^22^ For example, Yadav *et al.* studied PM, sulfur dioxide (SO_2_) and nitrogen oxide (NO_X_) ground level concentrations using air modeling with AERMOD model in an industrial area located about 20 km from Tumkur in Bangalore (India). They found maximum concentrations at 1770 m of NO_X_ at 24 μg/m^3^, of SO_2_ at 1.2 μg/m^3^ and for PM at 0.0028 μg/m^3^.^20^ Gibson *et al.* evaluated the concentration of PM_2.5_, NO_X_ and SO_2_ in a rural area (Nova Scotia) and urban area (Halifax) in Canada using the AERMOD Gaussian plume air dispersion model. Their findings showed that the pollutant concentrations in Nova Scotia were lower than in Halifax: 0.1< 2.5 μg/m^3^, 0.1 < 4.0 μg/m^3^ and 0.16 < 1.0 μg/m^3^ for PM_2.5_, NO_2_ and SO_2_, respectively.^21^ Amoatey *et al.* estimated the concentrations of SO_2_, NO_2_ and PM emitted in Tema Oil Refinery (Ghana) using AERMOD and CALPUFF models across seasons. The obtained maximum daily concentrations were higher during the heavy rainy season than minor rainy and dry seasons (37.7 μg/m^3^ for SO_2_, 9.6 μg/m^3^ for NO_2_, and 38.8 μg/m^3^ for PM_2.5_), respectively. 12,22

The National Road N2 of Togo, from Lomé to the Aného toll, covers a distance of 15.4 km and heavily trafficked by vehicles from the Gulf of Guinea countries. Particulate matter, SO_2_ and NOx, are the main pollutants from road traffic and industries. Few studies have been conducted on air quality and fuel quality in Togo. 15,23,24 It is thus necessary to evaluate the concentration of these pollutants in the air along the National Road N2. The results will be useful for the assessment of air pollution impacts on users, the health of nearby residents and the surrounding ecosystem using the most predictable modeling system, AERMOD.

The objective of the present study was to estimate the concentration of SO_2_, NO_x_ and PM emitted along the N2 in Togo in order to determine the pollution rate compared to international standards set by the WHO. This study could be useful for decision makers setting air quality policies for the future, in order to monitor the emissions of atmospheric pollutants, and for future studies as a baseline on the health of the population in the country.

## Methods

**Figure 1:**
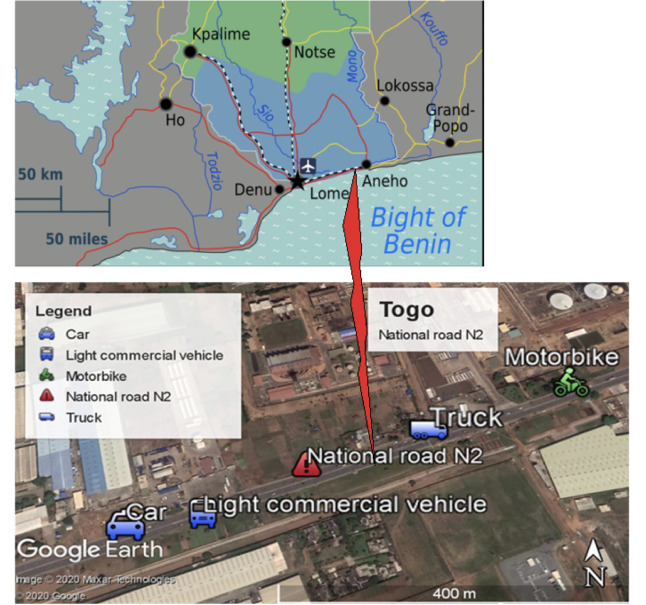
Road map of south Togo and the national road N2

This study was carried out on the national road of Togo, the National Road N2, from Lomé to the Aného toll, which travels over a distance of 15.4 km *(Figure 1).* The National Road N2 (Lomé-Aneho), about 50 km long, lies along the coast of Gulf of Guinea. The present case study focused on the evolution of air quality in the highly trafficked area for pollutants (SO_2_, NO_x_, and PM emissions). Figure 1 presents a road map of south Togo and a Google map photo of the study area along the National Road N2.

### Model set up

The Industrial Source Complex Short Term Version 3 model, which is included in the United States Environmental Protection Agency (USEPA) Regulatory Model, AERMOD View software, was used to perform the dispersion simulations of pollutants.^25^,^26^ The model was used to predict air concentrations and ambient impacts around the point/area and volume sources. The emission rates and the meteorological conditions were used as model inputs. Meteorological data, such as air temperature, wind speed, wind direction, ceiling height, cloud cover, pressure, relative humidity, and precipitation were obtained from the local station at Lomé (Togo) for the year 2018 along the National Road N2.^27^

**Figure 2:**
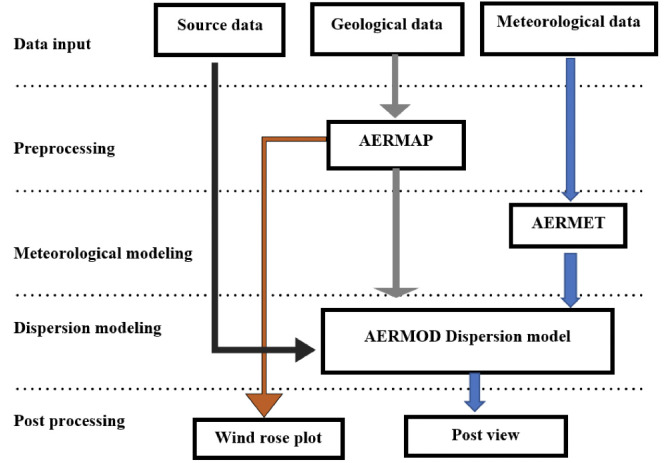
Data flow in the AERMOD modeling^20^

Figure 2 shows the flow and explains the processing of data during the detection of atmospheric pollutants in AERMOD.^20^

The model is composed of three parts as described by Yadav and coworkers: AERMOD meteorological preprocessor (AERMET) to extract meteorological data and to assess data quality; AERMOD terrain preprocessor (AERMAP) to merge all data available over a 24-hour period and record them in a single file; and AERMOD Gaussian plume model that reads the merged meteorological data and estimates the boundary layer parameters for the dispersion calculations.^20^,^26^ The main program was AERMOD, while data were pre-processed in AERMET and AERMAP.

Meteorological data such as wind speed, wind direction, temperature, and cloud cover were input to AERMET to determine the boundary layer parameters. AERMAP uses geological data to calculate the terrain height scale and to create receptor grids before passing receptor characteristics to AERMOD for final processing. Lastly, the source data were sent directly to AERMOD for processing. In addition, the wind rose plot for the most predominant wind direction and the pollutant contents were collected. The concentrations calculations are based on the following hypotheses: (i) the pollution sources were only the vehicles traveling along the selected road segment (total of 15.4 km); (ii) no other pollution sources were considered; (iii) the source was a line volume source with a width the same as the road (20 m) and a mixing layer height of 2 m. The concentration was calculated using the European Monitoring Evaluation Programme/European Environmental Agency air pollutant emission inventory guidebook, version 2016, considering the local traffic data and the highest uncertainty from the meteorological data. The original set of data contained daily average values and it was converted to hourly values in order to fit with the model.^27^ The concentration estimation methodology covered exhaust emissions of NO_x_, SO_2_, PM contained in the fuel. Nitrogen oxide emissions were further split into nitric oxide and NO_2_.

For exhaust emissions of NO_x_ and PM, the algorithm Tier 1 approach was used, following Equation 1. 28

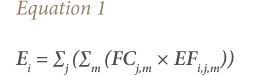
where, E_i_ is the emission of pollutant “i” (g); FC_j,m_ is the fuel consumption of vehicle category “j” using fuel “m” (kg); and EF_i,j,m_ is the fuel consumption-specific emission factor of pollutant “i” for vehicle category “j” and fuel “m” (g/kg).

Vehicles were categorized as passenger cars, light commercial vehicles, heavy-duty vehicles or L-category vehicles (which includes two or three wheelers, quadricycles and micro cars) and the considered fuels included petrol, diesel and natural gas. Tier 1 mean emission factors of NO_x_ and PM are presented in Table 1.^28^

**Table 1:** Tier 1 — Typical NOx and PM Content of Fuel^28^

**Category**	**Fuel**	**NO_x_ (g/kg fuel) mean**	**PM (g/kg fuel) mean**
Passenger car	Petrol	8.73	0.03
	Diesel	12.96	1.10
	Liquefied Petroleum Gas	15.20	0.00
Light Commercial Vehicle	Petrol	13.22	0.02
	Diesel	14.91	1.52
Heavy-duty vehicle	Diesel	33.37	0.94
	Compressed Natural Gas (Buses)	13.00	0.02
L- category (2/3 wheelers, quadricycles and micro cars)	Petrol	6.64	2.20

Examples of fuel sulfur content periods can be found in Table 2.

**Table 2:** Tier 1 - Typical Sulphur Content of Fuel (1 ppm = 10-6 g/g fuel)^28^

**Fuel**	**1996 base fuel**	**Fuel from 2000**	**Fuel from 2005**	**Fuel from 2009**
Petrol	165 ppm	130 ppm	40 ppm	5 ppm
Diesel	400 ppm	300 ppm	40 ppm	3 ppm

The emissions of SO_2_ per fuel-type *m* can be estimated by assuming that all sulfur in the fuel was transformed completely into SO_2_, using Equation 2.^28^

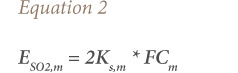
where, E_SO2,m_ is the emissions of SO_2_ per fuel “m” (g); K_s,m_ is the weight related to sulfur content in fuel of type “m” (g/g fuel); and FC_m_ is the fuel consumption of fuel “m” (g).

In the case of Togo, the sulfur content limit in 2018 is given in Table 3 from the Fuel Quality and Emission Standard Developments in Africa.^24^

**Table 3:** Sulfur Limits in Fuel^24^

**Fuel**	**Minimum**	**Maximum**	**Mean value**
Gasoline	501 ppm	3500 ppm	2000.5 ppm
Diesel	2001 ppm	10000 ppm	6000.5 ppm

Our investigations on transportation in Togo provide a characterization of traffic levels along the N2 *(Table 4).* We identified almost 10,000 vehicles in 24 hours.

**Table 4:** Vehicle type by time of day

Gasoline	Day	Night
Motorbike	1800	1580
Small car	1585	1450
Truck	130	145
Light commercial vehicle	450	345
Diesel	Day	Night
Small car	543	345
Truck	130	180
Light commercial vehicle	310	250

## Results

**Figure 3:**
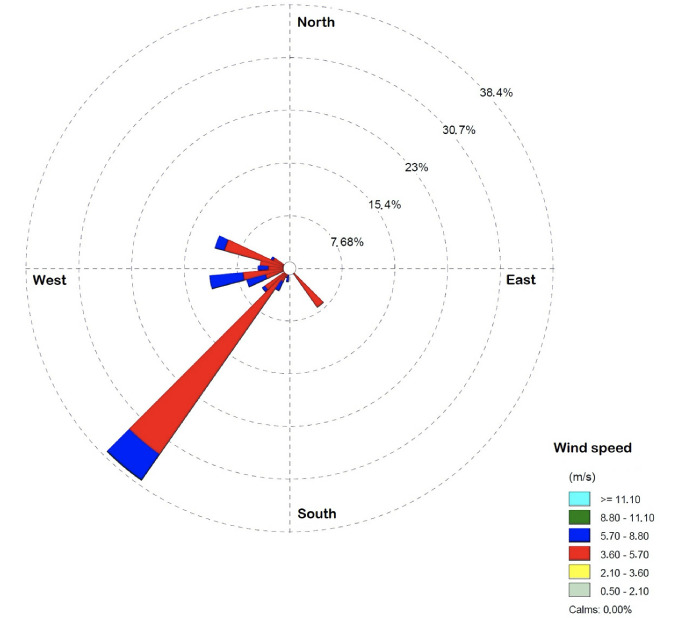
Wind rose of the meteorological station in the study domain

A wind rose diagram illustrates the speed, direction and frequency of winds of a given location using a center coordinate system. Meteorological pre-processed data were used to determine the corresponding wind rose plot *(Figure 3),* which shows the most predominant wind direction. The wind rose presented a main wind direction of south-west with an annual probability up to 38% and average wind speed between 3.6 – 5.7 m/s. Secondary directions were mainly west and northwest with a probability up to 12% and wind speed up to 8.8 m/s.

### AERMOD dispersion modeling results

Hourly, daily and annual averages of concentrations of pollutants (SO_2_, NO_x_, and PM) were investigated along the National Road N2, by AERMOD simulations. The maximal concentrations of emitted SO_2_, NO_x_ and PM obtained through AERMOD were compared with permissible limits of the WHO in Table 5.^29^

**Table 5:** Maximum Concentrations Obtained for NO_x_, SO_2_ and PM

**Pollutant gas**	**Time average**	**Maximum concentration [μg/m^3^]**	**WHO air quality guidelines, 2005 [μg/m^3^]^29^**
NO_x_	1 hour	16.78	200
	24 hours	9.89	-
	Annual	2.46	40
SO_2_	1 hour	71.91	-
	24 hours	42.41	20
	Annual	11.23	-
PM	1 hour	-	-
	24 hours	0.62	25
	Annual	0.15	10

### Concentration distribution of Nitrogen oxides

**Figure 4:**
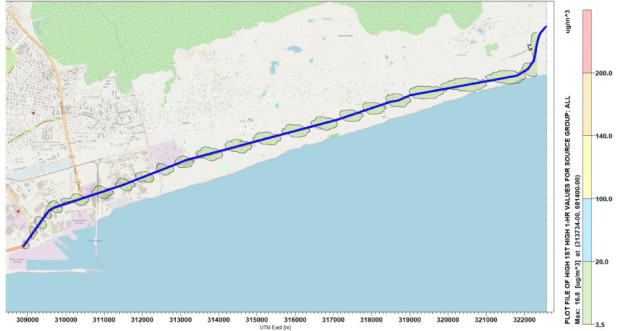
Maximum 1-hour average concentrations for NO_x_

**Figure 5:**
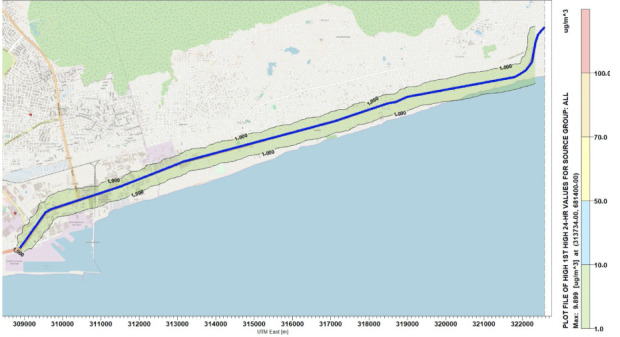
Maximum 24-hour average concentrations for NO_x_

**Figure 6:**
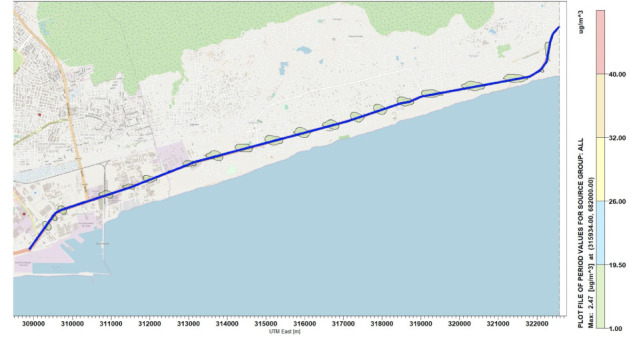
Maximum annual average concentrations for NO_x_

Simulations were performed for the concentration of NO_x_ along the National Road N2. Figures 4–6 present estimations of the maximum hourly, daily and annual concentrations of NOx on the National Road N2, respectively. The AERMOD simulation showed that the maximum hourly *(Figure 4)* and daily *(Figure 5)* average concentrations of NO_x_ were 16.78 μg/m^3^ and 9.89 μg/m^3^, respectively, at the position of the road where the Universal Transverse Mercator (UTM) coordinates were 313734 m E and 681400 m N. The maximum annual average concentration obtained on the road was 2.46 μg/m^3^ at the position of the road with the UTM coordinates of 315934 m E and 682000 m N *(Figure 6).*

### Concentration distribution of sulfur dioxide

**Figure 7:**
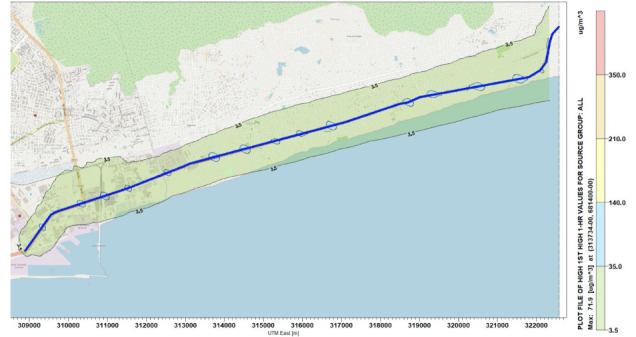
Maximum 1-hour average concentrations for SO_2_

**Figure 8:**
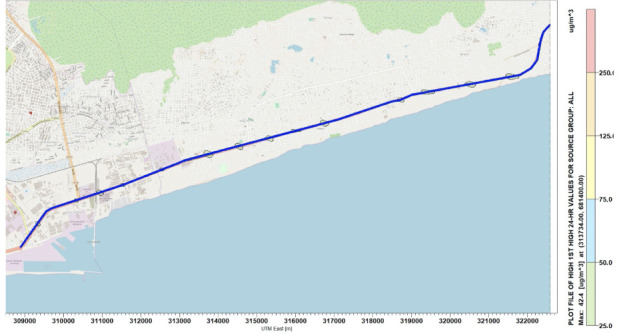
Maximum 24-hour average concentrations for SO_2_

**Figure 9:**
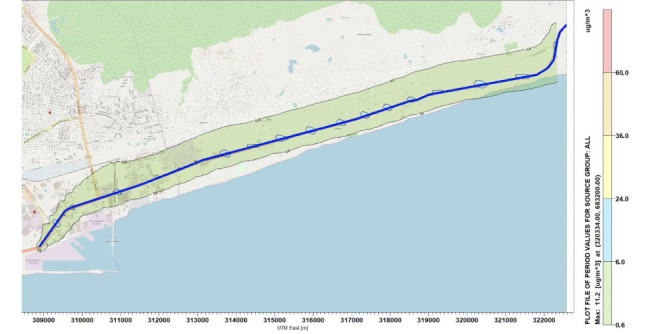
Maximum annual average concentrations for SO_2_

Concentration simulations performed for SO_2_ emitted on the National Road N2 are presented in Figures 7–9 for hourly, daily and annually concentrations, respectively. The maximum average concentrations of SO_2_ emitted on the road were 71.91 μg/m^3^ for hourly concentrations *(Table 5 and Figure 7),* 42.41 μg/m^3^ for daily concentrations (Figure 8) and 11.23 μg/m^3^ for annual concentrations *(Figure 9)* at the UTM coordinates of 313734 m E and 681400 m N.

### Concentration distribution of particulate matters

For PM, the model did not yield any valuable graphical representations, due to the very low obtained concentrations. In all AERMOD simulations, the PM concentration was low, as shown in Table 5, and hourly concentrations were below the detection level of the software. Daily and annual maximum average concentrations for PM, using AERMOD, were as low as 0.62 μg/m^3^ and 0.15 μg/m^3^, respectively, at the position of the road where UTM coordinates were 313734 m E and 681400 m N.

## Discussion

The maximum hourly concentration for the NO_x_ is lower than the permissible level of 200 μg/m^3^ recommended by the WHO.^29^ The annual maximum concentration for NO_x_ is also lower than the permissible level of 40 μg/m^3^ set by the WHO.^29^ Concentrations of NO_x_ on the National Road N2 according to AERMOD were much lower than the maximum permissible limits set by the WHO. These results are similar to the range of NO_2_ concentrations reported in other African cities, both hourly (9.4–135 μg/m^3^) and annually (2–175 μg/m^3^).^30^

The maximum daily concentration of SO_2_ exceeded twice the permissible limit set by the WHO at 20 μg/m^3^ and is in agreement with the daily concentration range of SO_2_ emitted in African cities (0.2–3662 μg/m^3^).^29^,^30^ However, this result is lower than air pollution daily data for SO_2_ from Cotonou (Benin) at the crossroads of the Dantokpa market, which were reported to be as high as 784.8–3662.4 μg/m^3^, and a reported concentration in Dakar of 68.54 μg/m^3^.^31^,^32^ Average levels of SO_2_ on the National Road N2 were higher than data obtained in Cairo (34 μg/m^3^) and in Bamako (29.03 μg/m^3^).^32^ In western African cities, lower concentrations of 0.5 μg/m^3^ was reported in Ouagadougou (Burkina Faso) and (3.66 μg/m^3^) in Abidjan (Côte d'Ivoire).^30^ Lower concentrations below the permissible limit set by the WHO were found in some African towns, such as Marrakesh (Morocco) and Tunis (Tunisia).^33^,^34^ These results should alert public authorities to monitor SO_2_ emissions to avoid environmental and health consequences. The main sources of SO_2_ are from traffic and combustion in motors, and reduction of sulfur levels in fuel in West African countries is crucial.

The daily and annual maximum average concentrations of PM were lower than the permissible limit set by the WHO, of 25 μg/m^3^ and 10 μg/m^3^, respectively.^29^ The PM concentration results from the present study were lower than those reported by Diallo *et al.* in the town of Lomé, where annual mean concentrations for PM_2.5_ ranged from 10.3–17.3 g/m^3^ and 11.6–18.4 g/m^3^ for PM_10_, which are lower than the permissible values of the WHO.^15^,^29^ Annual concentration levels of PM_2.5_ in Tema (Ghana) refinery were 12.6 μg/m^3^, much higher than the results found in the present study.^22^

Among the three studied pollutants emitted by engines on the National Road N2 in Togo, the concentration of SO_2_ was higher than the concentration of NO_2_ and PM and exceeded the recommended value set by the WHO.^29^ Sulfur dioxide is associated with engine exhaust from industries and traffic, and the results of the present study indicate that the fuels available in West African countries, especially in Togo and neighboring countries, are heavily sulfured. Atmospheric pollution is caused by human activities. High concentrations of air pollutants are associated with increased risk of human disease in cities. For example, Val *et al*. showed that exposure to particulate pollution could lead to adverse health effects such as the cancer in western Africa.^35^ The WHO reported that the premature death toll globally in 2016 due to air pollution was 4.2 million.^7^ Environmental problems such as climate change and global warming are related to the impacts of pollutants. The present study should prompt the government to make decisions on pollutant emissions, protecting the environment and human life. In addition, it is hoped that the present study could help to bring a greater awareness of the real impacts of pollution on human health and the environment

## Conclusions

The present study reported on the dispersion and concentrations of different pollutants such as SO_2_, NO_x_ and PM emitted on the National Road N2, in Togo, using AERMOD modelling with site-specific meteorological data. This road is heavily used by many types of vehicles traveling from Côte d'Ivoire, Ghana, Togo, Benin and Nigeria and adulterated fuels are regularly used, impacting human health and the environment. The results for these three pollutant gases showed that the hourly average concentration of SO_2_ was 71.91 μg/m^3^, 16.78 μg/m^3^ for NO_x_ and PM had very low and undetectable concentrations. Daily average gas concentrations were 42.41 μg/m^3^ for SO_2_, 9.89 μg/m^3^ for NO_x_ and 0.62 μg/m^3^ for PM. Results for annual concentrations were 11.23 μg/m^3^ for SO_2_, 2.46 μg/m^3^ for NO_X_ and 0.15 μg/m^3^ for PM. These results showed that concentrations of NO_x_ and PM were lower than the permissible limit of the WHO, similar to results in other African countries (9.4–135 μg/m^3^).^30^ However, the mean concentration of SO_2_ was almost twice the permissible limits set by the WHO (20 μg/m^3^).^30^ This illustrates the poor quality of fuel used in West African countries which may be very rich in sulfur. These results can assist in efforts by authorities to monitor pollutant levels in Togo. Efforts must be made to control the level of sulfur contained in fuels to avoid harmful impacts of sulfur dioxide on human health and the environment. We recommend point monitoring on this road by measuring gas pollutants concentrations and an assessment on impact to local population health.
